# The inhibitory effect of *Thymus vulgaris* extracts on the planktonic form and biofilm structures of six human pathogenic bacteria

**Published:** 2015

**Authors:** Zeinab Mohsenipour, Mehdi Hassanshahian

**Affiliations:** 1 *Department of Biology, Faculty of Sciences, Shahid Bahonar University of Kerman, Kerman, Iran*.

**Keywords:** *Biofilm*, *Thymus vulgaris*, *Antimicrobial effect*, *Pathogenic bacteria*

## Abstract

**Objective:**

Microorganisms are responsible for many problems in industry and medicine because of biofilm formation. Therefore, this study was aimed to examine the effect of *Thymus vulgaris* (*T. vulgaris*) extracts on the planktonic form and biofilm structures of six pathogenic bacteria.

**Materials and methods:**

Antimicrobial activities of the plant extracts against the planktonic form of the bacteria were determined using the disc diffusion method. MIC and MBC values were evaluated using macrobroth dilution technique. Anti-biofilm effects were assessed by microtiter plate method.

**Results:**

According to disc diffusion test (MIC and MBC), the ability of *Thymus vulgaris *(*T. vulgaris *) extracts for inhibition of bacteria in planktonic form was confirmed. In dealing with biofilm structures, the inhibitory effect of the extracts was directly correlated to their concentration. Except for the inhibition of biofilm formation, efficacy of each extract was independent from type of solvent.

**Conclusion:**

According to the potential of *Thymus vulgaris* (*T. vulgaris*) extracts to inhibit the test bacteria in planktonic and biofilm form, it can be suggested that *Thymus vulgaris* (*T. vulgaris*) extracts can be applied as antimicrobial agents against the pathogenic bacteria particularly in biofilm forms.

## Introduction

Biofilms are constituted by bacteria adhered onto surfaces, which, in turn, are surrounded by a matrix of organic polymers. They can be considered as a deposit where microorganisms are highly adhered onto a surface by means of appendixes of either protein or polysaccharide nature, referred to as glycocalyx (Criado, 1994 ▶). Biofilm-embedded bacteria are more resistant to antimicrobial agents and the immune defense system than their planktonic counterparts (Schlag et al., 2007 ▶). Hence, biofilms can cause significant problems in many areas, both in medical settings (e.g., persistent and recurrent infections, device-related infections) and in non-medical (industrial) settings (e.g., biofouling in drinking water distribution systems and food processing environments (Kumar and Anad, 1998 ▶; Flemming, 2002 ▶).

During the last two decades, the development of drug resistance as well as the appearance of undesirable side effects of certain antibiotics have led to the search for new antimicrobial agents mainly among plant extracts with the goal to discover new chemical structures to overcome the foregoing disadvantages (Srinivasan et al., 2001 ▶). Plants produce an enormous array of secondary metabolites and it is commonly accepted that a significant part of this chemical diversity serves to protect plants against microbial pathogens (Doxin, 2001 ▶). There are more than 35,000 plant species being used in various human cultures around the world for medicinal purpose (Srinivasan et al., 2001 ▶). Plant-derived drugs have been reported to be safe and without side-effects and antimicrobial properties of plant volatile oils have been recognized since antiquity (Cowan, 1999 ▶). More than 80% of the world's population relies on traditional medicine for their primary healthcare needs (Diallo et al., 1999 ▶).

*Thymus vulgaris *(*T. vulgaris*) or common thyme is a low growing herbaceous plant which sometimes becomes somewhat woody. It is native to southern Europe, where it is often cultivated as a culinary herb. It typically grows as a sub-shrub, between 15 and 20 cm tall (Al-Rawiand Chakravarty, 1988 ▶). *Thymus* species are considered as medicinal plants due to their pharmacological and biological properties. In native medicine, flowering parts and leaves of *Thymus* species have been extensively used as herbal tea, tonic, carminative, antitussive, and antiseptic as well as for treating colds (Rota et al., 2008 ▶). Thyme oil (common as *Thymus*) with a pungent odor and medical benefit has more than 44% phenols, which mainly consists of 41% Thymol and 3.6% Karvacrol, as confirmed by studies. The oil contains polyphenolic acids which are caffeic acid, triterpene, rosmaric acid, and oleanic acid, while resins, gums and tannins are about10% of the components of this plant. A as a result of anti-bacterial properties it is used as a disinfectant which is the main active ingredient in Listerine and toothpaste (Rizk, 1986 ▶).

The aim of this study was to evaluate antimicrobial activity of *T. vulgaris *extracts planktonic form and biofilm structures of six pathogenic bacteria. These bacteria were *Staphylococcus aureus*, *Bacilluscereus*,* Streptococcus pneumoniae, Pseudomonas aeruginosa, Escherichia coli *pathogenic serotype, and *Klebsiella pneumoniae*.

## Materials and methods

**Plant material isolation, identification, and extraction**

Fresh plants of *T. vulgaris* were collected in May, 2012, from Kerman, Iran. The taxonomic identities of the plant were confirmed by Dr. Mirtajaldini at the department of biological sciences, Shahid Bahonar University of Kerman, Kerman, Iran. The collected plants were washed under running tap water, air dried, and then ground into fine powder using an electric blender (Bosch Limited, Germany). Then, ten grams (10 g) of the powdered plant was dissolved in 100 ml of different solvents. The solvents used were ethanol 80% (Pars Chemic Co., Kerman, Iran) and methanol 96% (Pars Chemic Co., Kerman, Iran). *T. vulgaris *extracts were prepared using the maceration process for 30 h at 38^°C^ under constant shaking. Subsequently, the extracts were filtered through What man No.1 filter paper and the solvents were removed using rotatory evaporator apparatus. After wards, the obtained extracts were left at 40^°C^ for 24 h for complete dryness of each extract. Then, 40 mg of each dried extract was dissolved in appropriate volume of DMSO 1% so that concentration of sample reached40 mg/ml. Although some previous studies have shown that DMSO could have antibacterial effects, but in our study DMSO was used in nominal volume, because it was shown that it has no antimicrobial effect. These solutions were then filter sterilized with 0.22 µm mixed cellulose ester membranes (Millipore^ TM^, MA, USA). The extracts obtained were kept in sterile dark bottle at 4^°C^ for further use.

**Test bacteria and culture conditions**

Test microorganisms used in this study included six bacterial species, three Gram-positive (*S. aureus*, *B. cereus*in active form,and *S. pneumoniae*), and three Gram-negative (*P. aeruginosa, E. coli,* and* K. pneumoniae*). The tested microbial species were clinical isolates provided by the Faculty of Medicine, Department of Microbiology, Kerman University of Medical Sciences, Kerman, Iran.

The test microorganisms were maintained in NB/glycerol (20%) at 80^°C^. Nutrient agar (NA, Merck, Germany) containing Luria-Bertani (LB, Merck, Germany) was used to activate *S. pneumonia *while nutrient agar was used for other bacteria. The Mueller-Hinton agar (MHA, Merck, Germany) medium was used for disc diffusion assay and nutrient broth (NB, Merck, Germany) was used for the minimum inhibitory concentration (MIC) and the minimum bactericidal concentration (MBC) determination. 

The Mueller-Hinton agar was also used for the determination of the MBC on these species. 

The tryptic soy broth (TSB, Merck, Germany) medium was used for anti-biofilm assay. For all assessments for *S.* *pneumoniae*in, medium was enriched by increasing LB. 

**Sensitivity of bacteria to standard antibiotics**

Ciprofloxacin (Sigma, USA) (2 mg/ml) was used as reference antibiotics against bacterial species.Ciprofloxacin has bactericidal activity against awide range of gram positive and negative bacteria, and it is effective in low doses. Therefore, this antibiotic was used as positive control.

**Determination of antibacterial activity by disc diffusion technique**

The antimicrobial activity of alcoholic extracts of *T. vulgaris *was evaluated on test bacteria using disk diffusion method (Bauer et al., 1966 ▶). An overnight culture broth of each test bacteria was diluted to obtain initial inoculums of 10^8^ colony forming unit (CFU)/ml. Five hundred microliters (500 µl) of standardized inoculum was spread on MHA plates using sterile swabs.

In the following, sterile 6-mm blank paper disks (Padtan Teb Inc., Tehran, Iran), saturated with filter sterilized plant extract at the prepared concentration (40 mg/ml) for about 2 h were allowed to dry at 37°C for 5 h (Androw,2001 ▶). The two discs prepared in the same condition with only the corresponding volume of ethanol and methanol were used as negative control. Ciprofloxacin (2 mg/ml) was used as positive control. Each of the discs was placed on lawn cultures and then the plates were incubated at 37^°C^ for 24 h and the zones of inhibition were measured in mm.

**MIC and MBC determination**

Determination of the MIC was carried out using the macrobroth dilution method as recommended by the Clinical and Laboratory Standards Institute (Motamedi et al., 2010 ▶) using NB as the test medium. 

Overnight cultures of bacteria were diluted to yield a final concentration of 5×10^5^CFU/ml. The reconstituted extracts were serially diluted in two-fold in NB medium to obtain various concentrations of the stock (0.09-50 mg/ml) and were assayed against the test bacteria. In the following, 1 ml of standardized inoculum (5×10^5^CFU/ml) was added to 1ml of each extract concentration. Then, all tubes were incubated at 37^°C^ for 18 h and MIC was defined as the lowest concentration that was able to inhibit bacterial growth. Three control tubes were maintained for each test batch. These included tube containing extract and growth medium, tube containing the growth medium and inoculums, and tube containing the inoculum and ciprofloxacin (2 mg/ml).

MBC values were determined using sub-culturing 150 µl of bacterial suspension from the MIC tubes into MHA plates and then incubated at 37^°C^ for 18 h. After incubation, the concentration at which no growth was seen was recorded as the MBC.

**BATH – bacterial adherence to hydrocarbon test**

The ability of biofilm formation was evaluated on test bacteria using BATH method (Sedlackova et al., 2011 ▶). Overnight cultures of bacteria were diluted to 9 ml PBS, vortexed at constant speed, and the optical density adjusted to 0.2-0.3 at 600 nm (OD_1_). Then, 200 µl hexadecane was added to each tube, vortexed, and were incubated for 10 min at 30^°C^. Thereafter, the absorbance of the bottom aqueous layer of each bacterial suspension was determined at 600 nm (OD_2_). BATH was calculated using the ratio between the values of OD_1_ and OD_2_.

$\mathrm{BATH}=\frac{\mathrm{OD1}-\mathrm{OD2}}{\mathrm{OD1}}\times 100$

**Inhibition of biofilm formation**

Biofilm formation in polystyrene microtiter plates was assayed as described by O’Toole and Kolter (1998) ▶ with some modifications. One hundred µl of three different extract concentrations (12.5, 25, and 50 mg/ml) were pipetted into the wells of the microtiter plates. Then, an overnight culture of each bacterial species was diluted 1:100 with fresh TSB and 100 µl of these inoculums were added to each well. Thereafter, microtiter plates were incubated for 24h at 37^°C^. Three control wells were maintained for each test batch.

These included wells containing extract and growth medium (extract control),wells containing the growth medium and inoculum (negative control),wells containing ciprofloxacin (2 mg/ml) and inoculum (positive control), and wells containing the growth medium (media control).

The attached biofilm mass was quantified using crystal violet staining (Jabra-Rizk et al., 2006 ▶). After incubation, the media was aspirated and non-adherent cells were removed by washing the wells three times with sterile phosphate buffer saline (PBS). In order to fix the adherent cells, 150 µl of methanol 96% was added to each well for 15 min. The microtiter plates were then stained with 200 µl of crystal violet 1% (Gram color-staining set for microscopy, Merck, Germany) for 20 minand excess stain rinsed off with running tap water.

The plates were air-dried and the CV bound to adherent cells was re-solubilized with 160 µl of 33% glacial acetic acid per well. The absorbance of each well was monitored with a microtiter plate reader (ELX-800, Biotec, India) at 630 nm. Percent inhibition of biofilm formation was calculated using the ratio between the values of OD_630nm_ wells with and without the extracts.

%inhibitionOD negative control-OD media control-OD test-OD extract controlOD negative control-OD media control×100

**Disruption of established biofilm**

Disruption of established biofilm structures was measured as described by Sandasi (2008) ▶ with some modifications. At first, biofilms were established in the microtiter plates by growing 100 μl of the standard bacterial culture (OD 600=0.2) for 24 h at 37^°C^. After incubation, the medium were aspirated and the planktonic cells were removed by washing the biofilms three times with sterile PBS. There after, three different concentrations (12.5-50 mg/ml) of *T. vulgaris* extracts was added to each well and plates were then placed back into the 37°C for 24 h.

The control wells were the same as those described above. The percentages of biofilm eradication in the presence of different concentrations of extracts were calculated employing the formula as described earlier. 

**Efficiency of extracts on biofilm metabolic activity**

The effect of *T. vulgaris* on the vitality of the selected bacteria was tested as reported by Ramage and Lopez-Ribot (2005) ▶. Initially, pre-formed biofilms were washed twice with PBS, three different concentrations (12.5-50 mg/ml) of *T. vulgaris* extracts were added and biofilms were incubated for an additional 24 h at 37^°C^. After wards, 50 µl of a triphenyl tetrazoliumchloride (TTC, Merck, Germany) solution was added allowing the reaction to occur in the dark at 37^°C^ for 3 h. TTC reduction was also measured with a microplate reader at 490 nm. The percentages of reduction of biofilm metabolic activity in the presence of different concentrations of extracts were calculated employing the formula as described earlier.

**Statistical analysis**

All experiments were conducted in triplicates. Data were analyzed statistically by determination of significant difference using SPSS version 18.0 for Windows and compared using analysis of variance (ANOVA) test. All tests were analyzed at the significance level p<0.05.

## Results

**Inhibitory efficiency of** ***T. vulgaris*** **extracts against planktonic form of pathogenic bacteria**

**Figure 1:**
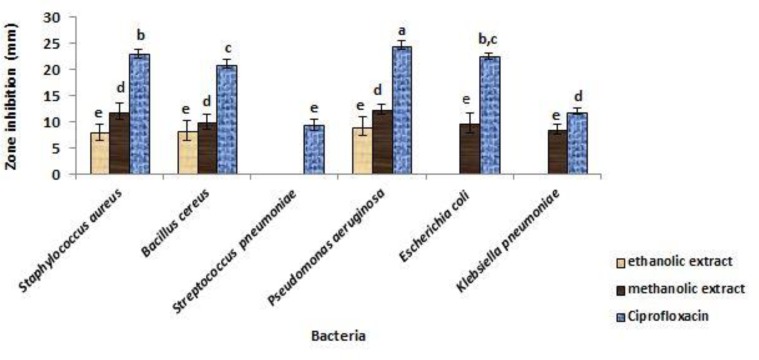
Antibacterial activity of *T. vulgaris* alcoholic extracts against test microorganisms using disc diffusion method (zone of inhibition in mm).Different letters indicate statistically significant differences (p<0.05

Figure 1 shows the zones of inhibition (ZOI) of *T. vulgaris* extracts on tested clinical bacterial pathogens. MIC and MBC values of these extracts are illustrated in Table 1.

**Table 1:** *T. vulgaris*minimal inhibitory concentrations (MICs) and minimal bactericidal concentrations (MBCs) of test bacteria.

**Bacteria**	**MIC Methanolic extract (mg/ml)**	**MIC Ethanolic extract (mg/ml)**	**MBC Methanolic extract (mg/ml)**	**MBC Ethanolic extract (mg/ml)**
*Staphylococcusaureus*	0.312	0.625	1.25	2.5
*Bacilluscereus*	0.625	1.25	2.5	5
*Streptococcus pneumoniae*	0.156	0.312	0.625	1.25
*Pseudomonas aeruginosa*	0.156	0.625	0.312	2.5
*Escherichia coli*	1.25	2.5	5	10
*Klebsiellapneumoniae*	0.625	1.25	1.25	5

In disc diffusion analysis,* T. vulgaris* extracts inhibited all tested bacteria properly in 1% significant level (p<0.01). However, the inhibitory effects of these extracts on *S. pneumoniae* and *K. pneumonia* were not significant at 5% level (p=0.058). The biggest ZOI in disk diffusion experiment was observed on *P. aeruoginosa*, however, *T. vulgaris* extracts did not show any ability to produce ZOI against *S. pneumonia.* Moreover, ethanolic extract of this plant was not effective against *E. coli *and *K. pneumoniae*. MIC values of these extracts were highest for *E. coli *and lowest for *S. pneumonia* between all tested bacteria.

Considering that the extracts in broth media in MIC test, in the lower concentration which was used in preparing disks (0.156-2.5 mg/ml) could inhibit all tested pathogenic bacteria, it can be concluded that the inhibitory efficiency of these extracts in broth medium is more than the solid medium.

**The ability of bacteria to attach the surfaces**

The percentage expression of bacterial affinity to hydrocarbon phase in BATH test is shown in Table 2. Hydrophobicity is considered critical for initiating bacterial adhesion interactions, therefore, the BATH value indicates the cell surface hydrophobicity and the ability of biofilm formation. The highest BATH value was observed for *S. pneumoniae* (48.02%) and the lowest value observed for *B. cereus* (6.25%).

**The inhibitory effects of** ***T. vulgaris*** **extracts against biofilm structures**

**Figure 2:**
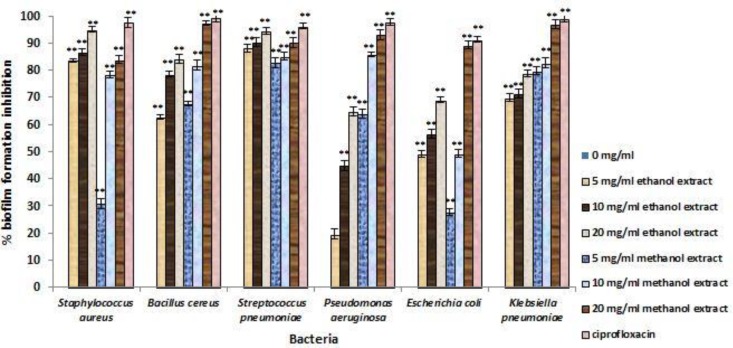
Percent reduction of biofilm formation for test bacteria treated with different concentrations of *T. vulgaris* for 24 h. * Differences between control (no inhibition) and treatment with extracts (* p < 0.05, ** p < 0.001).

**Figure 3:**
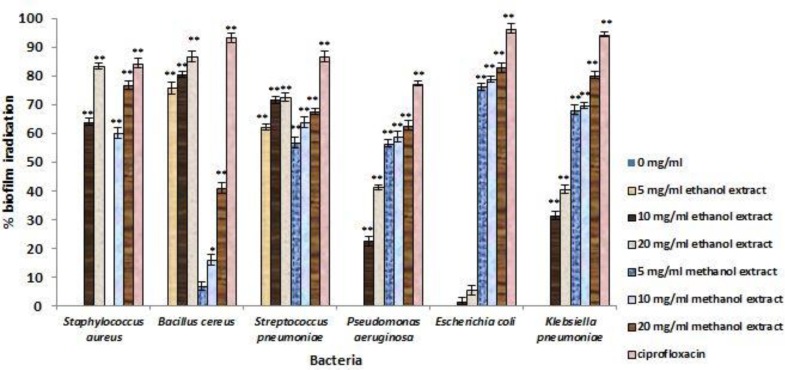
Percent disruption of biofilm formation for test bacteria treated with different concentrations of *T. vulgaris* for 24 h. * Differences between control (no inhibition) and treatment with extracts (* p < 0.05, ** p < 0.001).

**Figure 4:**
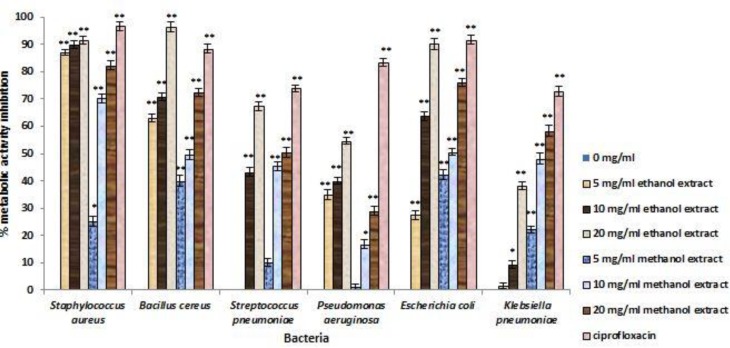
Percent reduction of biofilm metabolic activity for test bacteria treated with different concentrations of *T. vulgaris* for 24 h. * Differences between control (no inhibition) and treatment with extracts (* p < 0.05, ** p < 0.001).

The inhibitory efficiency of each concentration of* T. vulgaris* extract on preventing biofilm formation, demolishing biofilm structures, and inhibition of metabolic activity of biofilm are shown in Figures 2, 3, and 4. According to the F value of the ANOVA analysis on tested data, it was confirmed that inhibitory efficiencies of* T. vulgaris* extracts were significant at 1% level (p<0.01).

**Table .2:** The BATH percentage of test bacteria

***Klebsiellapneumoniae***	***Escherichia coli***	***Pseudomonas aeruginosa***	***Streptococcus pneumoniae***	***Bacilluscereus***	***Staphylococcusaureus***	**Bacteria**
23.8	10.27	27	48.02	6.25	42.63	BATH percentage

Based on these results, it was concluded that type of bacteria, type of solvent, and concentration of extracts were significant on inhibitory effect of *T. vulgaris* extracts on biofilm structures (p<0.01). However, the inhibition of biofilm formation in the treatment with *T. vulgaris* extracts were independent from type of solvent (p=0.456). the concentration of each extract showed linear correlation with inhibitory effect, thus, the inhibitory effect increases with increasing concentration. According to the value of mean of inhibitory effect of selected concentration of *T. vulgaris *extracts, these extracts had the ability to inhibit 50% of biofilm formation in tested bacteria. The highest inhibition of biofilm formation was observed against *S. pneumoniae* (88.51%) and the lowest inhibition observed for biofilm formation of *E. coli *(56.83%). 

For destruction of biofilm structures, biofilm of *S.pneumoniae* were the most sensitive structure (65.82 %) and biofilm of tested Gram negative bacteria showed low sensitivity, however, the mean value of destruction for these bacteria was 43.15%. Metabolic activity of bacteria in biofilms treated with the* T. vulgaris* extract had remarkable decrease. The greatest reduction was observed in *S. aureus* biofilm (74.47%) and the lowest reduction observed in *P. aeruginosa* (29.38%) and *K. pneumonia* biofilms (29.36%).

## Discussion

The importance of biofilm formation in the development of drug resistance among pathogenic bacteria has been well documented. The presence of these microbial communities is often associated with various chronic diseases and eradication of these communities is rarely achieved, with dire consequences for patients (Parsek and Singh, 2003 ▶; McCann et al., 2008 ▶). Novel strategies are therefore required to deal with these biofilm-mediated infections (Doxin,2001 ▶). Therefore, the present study evaluated the antibacterial activity of *T. vulgaris* alcoholic extracts.

Disc diffusion analysis show that* T. vulgaris* extracts had high ability to inhibit the growth of *P. aeruginosa* and *S. aureus. *However, these extracts had low inhibition efficiency on *E. coli *and *B. ceruse* and did not show inhibitory effect on other tested clinical bacterial pathogens. *T. vulgaris* extracts inhibited the growth of all tested bacteria in broth media with low concentration which was used in solid media. According to these results, it can be concluded that antimicrobial compounds in *T. vulgaris* extracts similar to other plant extracts have low diffusion in solid media compared with broth media. Therefore, for favorable impact on solid media, much higher concentration than broth media is needed.

The MIC values for* T. vulgaris *extracts on tested bacteria were in 0.156 to 2.5 concentration range which confirmed the inhibitory ability of these plant extracts. Moreover, higher values of MBC than MIC indicated the bacteriostatic properties of* T. vulgaris* extracts.

*T. vulgaris* extracts were efficient in dealing with biofilm structures*. *The inhibitory effect of these extracts was directly correlated to concentration and except on inhibition of biofilm formation, the type of solvent were efficient on the anti-biofilm ability of each extract. The ability of* T. vulgaris* ethanolic extract in inhibition of biofilm formation of *S. aureus* and *E. coli* did not show any significant difference compared to inhibition of metabolic activity of microbial cell in the biofilm structure, but was more effective than destruction of biofilms of these bacteria. The effect of *T. vulgaris* extracts on biofilm formation of other tested bacteria was more pronounced than the ability of these extracts to destroy biofilms and inhibit the metabolic activity. Considering that the active components of *T. vulgaris* and the inhibitory mechanisms on biofilm structures were not investigated in this research, it can be suggested by characterization of these compounds, it will be possible to interpret the difference inhibitory effect of these extracts between various tested clinical bacteria. 

Some researcher confirmed antimicrobial properties of *T. vulgaris* against different microorganisms such as* S. aureus*, *E. coli*, *Listeria monocytogenes*, *Streptococcus pyogenes*, *S. pneumoniae*, *K. pneumoniae*, *Bacillus subtilis*, *Aspergillus niger, *and* Candida albicans*. These researches showed that inhibitory effect of thyme essential oil was more than extracts of this plant and with increasing of concentration, the antimicrobial properties was enhanced (Dobre et al., 2011 ▶; Ismail et al., 2012 ▶; Mohsenzadeh, 2007 ▶; Priti, 2012 ▶). Our results in the present study are in agreement with other researchers. Therefore, It can be concluded that the *Thymus* extract inhibitory effect, similar to other antimicrobial compounds, is directly correlated to concentration.

Kavanaugh and Ribbeck (2012) ▶ showed that disks impregnated with thyme essential oil had efficient inhibitory effect on planktonic form of *S. aureus*and* P. aeruginosa.* Moreover, these essential oils remarkably decreased the biofilm formation by these bacteria as in 0.2% concentration destroyed living bacteria in the biofilm. Their research confirmed that the inhibitory effect of red thyme essential oil was more efficient than some antibiotics such as ampicillin, of loxacin, and gentamicin. The study carried out by Gonçalves et al. (2011) ▶ confirmed the considerable inhibitory effect of *T. vulgaris *extracts against *S. mutans*. Their research showed that the inhibitory effect of these extracts was higher when these extracts dissolved in ethanol compared tomineral oil. This may be due to the ability of ethanol to dissolve the polar compounds compared to mineral oil.Therefore, ethanol is a better choice to reach the thyme extracts with higher antimicrobial efficiency.

Sandasi (2008) ▶ reported the inhibitory effect of *T. vulgaris* extract against planktonic form of *C. albicans*, *L. monocytogense,* and *P. aeruginosa*. The inhibitory effect of alcoholic extract was more than aqueous extract. This study showed that *T. vulgaris *extract could inhibit attachment of these mentioned bacteria, however the destruction of biofilm by these extracts was lesser than inhibition of biofilm formation.

According to the results of this research and other studies that have studied different species* T. vulgaris,* the antimicrobial potential of this plant is confirmed and the extractions of this plant are suitable choices against pathogenic microorganisms. Hence, further investigations in order to identify and purifyactive components of these extracts and also to understand their mechanism of action on biofilm structures in order to achieve a good source of antimicrobial agent with pathogenic microorganisms are recommended.
